# Strain-Specific Virulence Phenotypes of *Streptococcus pneumoniae* Assessed Using the *Chinchilla laniger* Model of Otitis Media

**DOI:** 10.1371/journal.pone.0001969

**Published:** 2008-04-09

**Authors:** Michael L. Forbes, Edward Horsey, N. Luisa Hiller, Farrel J. Buchinsky, Jay D. Hayes, James M. Compliment, Todd Hillman, Suzanne Ezzo, Kai Shen, Randy Keefe, Karen Barbadora, J. Christopher Post, Fen Ze Hu, Garth D. Ehrlich

**Affiliations:** 1 Department of Pediatrics, Allegheny General Hospital, Pittsburgh, Pennsylvania, United States of America; 2 Center for Genomic Sciences, Allegheny General Hospital-Allegheny-Singer Research Institute, Pittsburgh, Pennsylvania, United States of America; 3 Department of Surgery, Allegheny General Hospital, Pittsburgh, Pennsylvania, United States of America; 4 Department of Microbiology and Immunology, Drexel University College of Medicine, Pittsburgh, Pennsylvania, United States of America; 5 Department of Neurosurgery, Allegheny General Hospital, Pittsburgh, Pennsylvania, United States of America; 6 Department of Pediatrics, Children's Hospital of Pittsburgh, Pittsburgh, Pennsylvania, United States of America; Centre for DNA Fingerprinting and Diagnostics, India

## Abstract

**Background:**

*Streptococcus pneumoniae* [*Sp*] infection is associated with local and systemic disease. Our current understanding of the differential contributions of genetic strain variation, serotype, and host response to disease phenotype is incomplete. Using the chinchilla model of otitis media [OM] we investigated the disease phenotype generated by the laboratory strain TIGR4 and each of thirteen clinical strains (BS68-75, BS290, BS291, BS293, BS436 and BS437); eleven of the thirteen strains have been genomically sequenced.

**Methodology/Principal Findings:**

For each strain 100 colony forming units were injected bilaterally into the tympanic bullae of 6 young adult chinchillas under general anesthesia. All animals were examined daily for local and systemic disease by a blinded observer. Pneumatic otoscopy was used to evaluate local disease, and behavioral assessments served as the measure of systemic disease. Virulence scoring was performed using a 4-point scale to assess four clinical parameters [severity and rapidity of local disease onset; and severity and rapidity of systemic disease onset] during a 10-day evaluation period. Highly significant variation was observed among the strains in their ability to cause disease and moribundity.

**Conclusions/Significance:**

As expected, there was a significant correlation between the rapidity of systemic disease onset and severity of systemic disease; however, there was little correlation between the severity of otoscopic changes and severity of systemic disease. Importantly, it was observed that different strains of the same serotype produced as broad an array of disease phenotypes as did strains of different serotypes. We attribute these phenotypic differences among the strains to the high degree of genomic plasticity that we have previously documented.

## Introduction

Otitis media (OM) is the most common malady for which children receive medical and surgical attention in the United States and Europe [1]. Nearly all children will experience an episode of OM during the first two years of life, and approximately 2 million children receive myringotomies and ventilation tubes annually with a total treatment cost of nearly five billion dollars [2]–[5]. OM has a wide range of clinical presentations [1] and if untreated can, in rare cases, lead to invasive and systemic disease states that are associated with moribundity. OM pathogenesis is multifactorial with pathogen-specific virulence factors, and the host's anatomy, immune status, and genetics all playing roles in phenotypic expression [6]–[15]. Recent evidence has established that genetic polymorphisms in the promoter of the human IL-9 gene result in its over expression in response to endotoxin exposure resulting in increased mortality [8]. Other studies [16]–[19] have illuminated the complexities of pathogen-host interactions and provided evidence supporting the hypotheses that intrinsic pathogen virulence factors, as well as specific elements of the host response make independent contributions to the development of clinical phenotype providing potential targets for future therapies [20], [21], [22].

These investigations continue to yield provocative answers and questions, but the local and systemic clinical challenges associated with OM remain. A uniformly predictive way of stratifying risk in persons with OM that provides for the reliable identification of persons with self-limited disease or chronic local disease (otitis media with effusion [OME]) from those with early invasive disease has been elusive. This difficulty in differentiation exists because the initial disease presentation for both self-limited, and invasive infections, are similar.

*S. pneumoniae* is one of the three bacterial pathogens (together with *Haemophilus influenzae* and *Moraxella catarrhalis* ) most frequently identified by culture and molecular diagnostics from middle-ear effusions obtained from children with acute and chronic forms of OM [23]. This pathogen is also associated with a number of other important infections including pneumonia, meningitis, bacteremia and osteomyelitis [24]–[27]. While over 90 different serotypes of *Sp* are known, less than 30 are responsible for over 90% of human disease [28]. This suggests that serotype is important, but not the sole determinant of pathogenicity.

In a survey study Shen et al. [29] demonstrated that each of ten clinical pneumococcal strains varied substantially from each other in terms of gene content providing support for the distributed genome hypothesis (DGH) [30]. This study suggested that each strain contains a unique distribution of genes from a population-based supragenome [29] that is much larger than the genome of any single strain. As a direct test of the DGH, Hiller et al. showed in an exhaustive pair-wise comparison of 17 complete pneumococcal genomes that on average each clinical strain pair varied (i.e. the gene was present in one strain and absent in the other) at 18% of their genic loci [31]. Moreover, this study documented that between 21% and 33% of the genes in each strain are non-core, or distributed genes. Thus, each strain contains a unique distribution of genes, not alleles, from a population-based supragenome, which is far greater in size than the genome of any given strain. This observation of extensive genomic plasticity driven by active horizontal gene transfer mechanisms has been widely observed among other pharyngeal pathogens as well, including *Haemophilus influenzae, Neisseria meningiditis,* and *Streptococcus agalactiae* [29], [32], [33], [34].

Previous animal studies of OM using the *Chinchilla laniger* (chinchilla) model suggested that this model could differentiate between both different bacterial species and different clinical strains of a single species with respect to the severity of both local and systemic disease [35], [36], [37]. The gram-negative bacterium *Haemophilus influenzae* often yielded lethal, systemic disease characteristic of fulminant endotoxemia, whereas inoculation with *Sp* produced an unpredictable variety of apparently strain-specific clinical phenotypes.

The objective of this study was to determine if each of fourteen genetically characterized *Sp* clinical strains produced a consistent and unique disease phenotype with respect to type (local or systemic), rapidity of onset, and severity of disease following middle-ear inoculation. Future studies will examine the discrete phenotype/genotype relationships of these strains.

## Results

### Study Design

**Figure 1:**
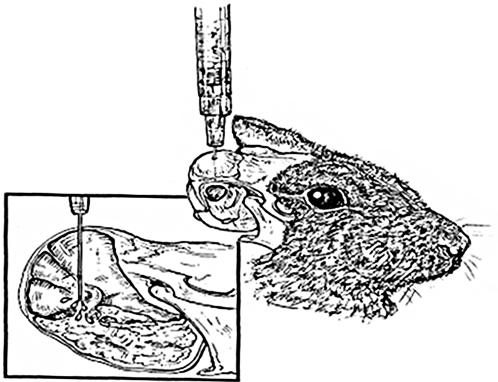
The transbullar technique is used to induce *S. pneumoniae* otitis media in the chinchilla under general anesthesia.

We undertook a comparative evaluation of the disease phenotypes induced by 14 genotypically distinct pneumococcal strains [29], [31], including 5 strains of serotype 9 and 2 strains of serotype 6, and one unencapsulated strain that was known to cause localized disease using the chinchilla model of OM (Fig. 1). Strains will be referred to by their name followed by the serotype within parenthesis. The rapidity and severity of both otoscopic and systemic clinical signs for each strain were evaluated on a 4-point scale using a cohort of 6 animals by a blinded observer (Table 1).

**Table 1:** Scoring System to Quantify Streptococcus Pneumoniae Pathogenicity in the Chinchilla

Otologic Score	0	1	2	3	4	
Degree of otoscopic disease	none	Mild	Moderate	Frank purulence	Tympanic membrane rupture	
Systemic Score	0	0.5	1	2	3	4
Systemic Disease	normal	Slightly lethargic Upright and steady on feet Immediately responds to stimulation by actively moving around cage Eating and drinking	Slightly lethargic Upright and steady on feet Head and ears down Responds by actively moving around cage rapidly when stimulated by voice or touch Eating and drinking Eyes ½ open	Moderately lethargic Slightly ataxic Able to keep self upright Moving around cage slowly only when stimulated by voice or touch Eating treats and drinking water	Same as 2 but not eating or drinking	Extremely lethargic Extremely ataxic Not able to maintain an upright position Barely moving around cage Dyspnea Considered Moribund

### Otoscopic Signs

**Figure 2:**
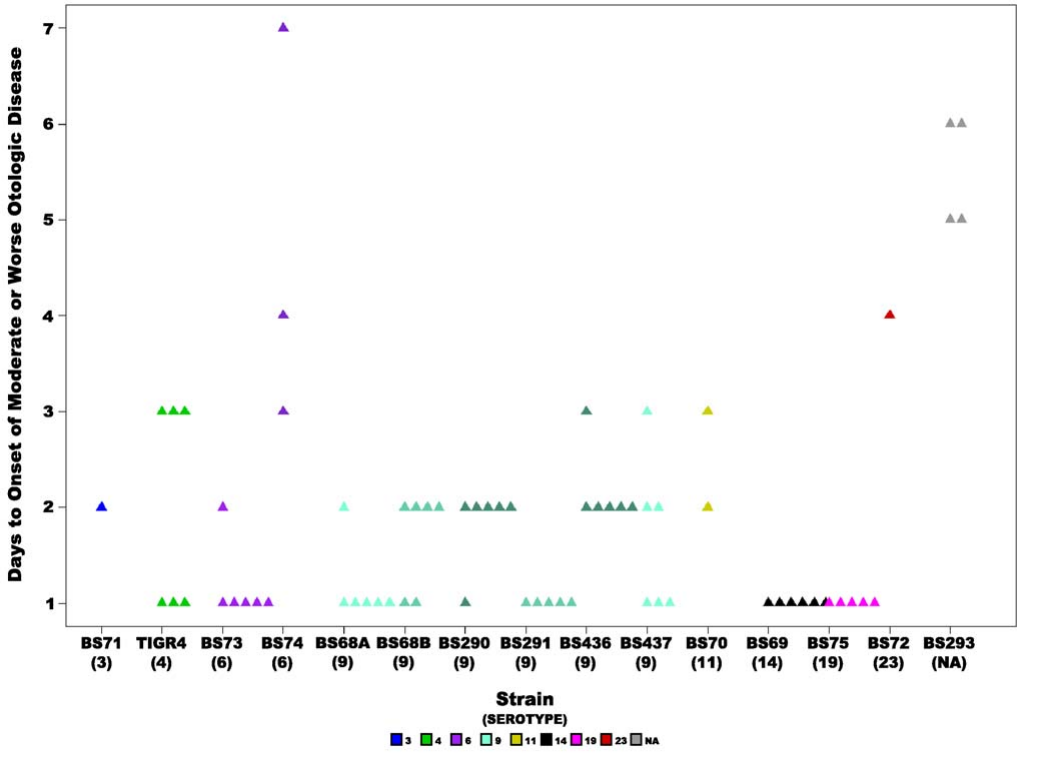
Rapidity of otologic disease onset. Scatter plot showing the number of days it took for chinchillas inoculated with each of the *S. pneumoniae* clinical strains to develop moderate or worse (score ≥2) otologic disease, where each triangle represents a single animal. Statistical analysis of all the strains revealed a significant difference, the p-value for the ANOVA analysis was 9.6e^−15^, and for the Kruskal-Wallis test was 2.4e^−5^. Animals are not included that did not develop local disease before the end of the experiment or before reaching a moribund state. Colors represent different serotypes, and shades of the same color different strains of the same serotype or in the case of BS68(9) two cohorts from different experiments. NA: non applicable.

Across the fourteen strains the rapidity of otoscopic changes, defined as the days to the development of unambiguous otological signs (a score of 2 or above since differences between scores of 0 and 1 were difficult to discern, Table 1), produced a mean of 1.93 days±1.35 (standard deviation, SD) (Fig. 2). Note, that animals that never developed otoscopic disease (such as all PBS controls) were excluded from this set. An ANOVA analysis of the differences among the strains produced highly significant results (p-value = 9.6e^−15^) as did repetition of the analysis by the non-parametric Kruskal-Wallis test (p-value = 2.4e^−5^) (Table 2). The means for the individual strain cohorts ranged from 1 day for strains BS291(9), BS75(19) and BS69(14) to 5.5 days for the unencapsulated strain BS293 and 4.6 for the capsulated strain BS74(6) (Fig. 2, Table 3). Statistical analysis of all the strain pairs using Tukey HSD showed that unencapsulated BS293 and BS74(6), the strains with slowest onset of disease, differed significantly from strains BS290(9), BS291(9), BS436(9), BS437(9), BS68(9), BS69(14), BS70(11), BS73(6), BS75(19), and TIGR4(4). In addition unencapsulated BS293 also differed from BS71(3). Finally BS72(23) differed significantly from BS291(9), BS68(9), BS69(14), BS73(6) and BS75(19), the strains with the fastest onset of disease.

**Table 2:** Summary of All Statistical Analysis

Group	Parameter	ANOVA	Kruskal-Wallis	Kaplan-Meier	Fisher-Exact
All	Otoscopic Rapidity	9.60E-15	2.42E-05	6.44E-12	N/A
All	Otoscopic Severity	9.56E-05	0.00118	N/A	N/A
All	Systemic Rapidity	0.09392	0.01637	N/A	N/A
All	Systemic Rapidity 2*	0.03066	0.001187	N/A	N/A
All	Systemic Severity	2.69E-09	4.22E-06	N/A	N/A
All	Moribundity	N/A	N/A	N/A	3.90E-07
Capsulated	Systemic Rapidity	0.106	0.02055	N/A	N/A
Capsulated	Systemic Rapidity 2*	0.03066	0.001187	N/A	N/A
Capsulated	Systemic Severity	1.22E-05	0.000171	N/A	N/A
Capsulated	Moribundity	N/A	N/A	N/A	0.000117
Serotype 9	Otoscopic Rapidity	0.007666	0.01092	N/A	N/A
Serotype 9	Otoscopic Severity	0.3475	0.4509	N/A	N/A
Serotype 9	Systemic Rapidity	0.1116	0.04126	N/A	N/A
Serotype 9	Systemic Rapidity 2*	0.001299	0.001762	N/A	N/A
Serotype 9	Systemic Severity	1.77E-01	1.84E-01	N/A	N/A

* Systemic Rapidity 2 measured onset to moderate or worst systemic disease (score ≥2)

N/A: not applicable.

**Table 3:** Averages and standard deviations observed within each strain for local and systemic disease

Parameter	BS68	BS290	BS291	BS436	BS437	BS293	BS73
otoscopic rapidity (days)	1.417±0.515	1.833±0.408	1±0	2.167±0.408	1.667±0.816	5.5±0.577	1.167±0.408
otoscope severity	2.583±0.669	2.167±0.408	2.2±0.447	2.333±0.516	2.833±0.983	1.667±0.516	2.833±0.408
rapidity to any systemic disease (days)	1.583±0.515	4±3.95	2±0	3±1.414	2.8±0.447	2±0	3.333±2.875
rapidity to moderate or worse systemic disease (days)	2.091±0.539	3.333±0.577	2.8±0.447	3.6±0.894	4±1.414	NA	3.5±2.739
systemic severity	3.75±0.866	2.25±1.917	4±0	3.333±1.633	2.75±1.943	0.5±0	3.667±0.816

Parameter	BS75	BS69	TIGR4	BS71	BS72	BS70	BS74
otoscopic rapidity (days)	1±0	1±0	2±1.095	2	4	2.5±0.707	4.667±2.082
otoscope severity	2.833±0.983	2.833±0.408	2.833±0.753	1.333±0.816	1.333±0.816	1.667±1.033	1.833±0.983
rapidity to any systemic disease (days)	1.667±0.516	3±1.673	3±0	1.5±0.548	1.667±0.516	1.667±0.816	2.5±1
rapidity to moderate or worse systemic disease (days)	1.833±0.408	3±1.673	3±0	1.5±0.548	2.167±1.472	1.833±1.169	2.5±1
systemic severity	4±0	3.667±0.516	0.667±1.033	4±0	4±0	4±0	2.5±1.975

N/A: non-applicable.

**Figure 3:**
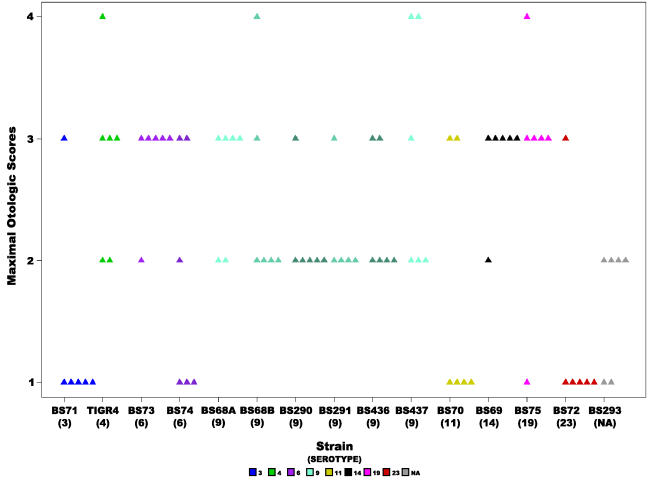
Severity of otoscopic change. Scatter plot illustrating the maximal otologic score for each animal inoculated with each of the *S. pneumoniae* strains, where each triangle represents a single animal. Statistical analysis of all the strains revealed a significant difference (ANOVA p-value = 9.56e^−5^ and Kruskal-Wallis p-value =  0.00118). Colors represent different serotypes, and shades of the same color different strains of the same serotype or in the case of BS68(9) two cohorts from different experiments. NA: non applicable.

We also analyzed the maximal otologic score for each animal as a measure for the severity of local disease (where scores of 0 and 1 were considered to be the same since they are hard to discern) (Fig. 3). The mean score for maximal otologic disease among the fourteen strains was 2.26±0.87. Mean scores for individual strains ranged from 1.3 for strains BS71(3) and BS72(23) to 2.83 for strains BS75(19), BS437(9), BS73(6), BS69(14), and TIGR4(4) (Table 3). Statistical analysis of this data set showed a significant difference amongst the strains (ANOVA p-value = 9.56e^−5^, and Kruskal-Wallis p-value =  0.00118) (Table 2). Further analysis of all the strain pairs using Tukey HSD, showed that the strains with the least severe otoscopic disease BS71(3) and BS72(23) differed significantly from BS437(9), BS69(14), BS73(6), BS75-19, and TIGR4(4). There was a significant correlation between days to onset of otoscopic disease and the maximal otoscopic score (Spearman's rank correlation rho = −0.311 with p-value = 0.009).

**Figure 4:**
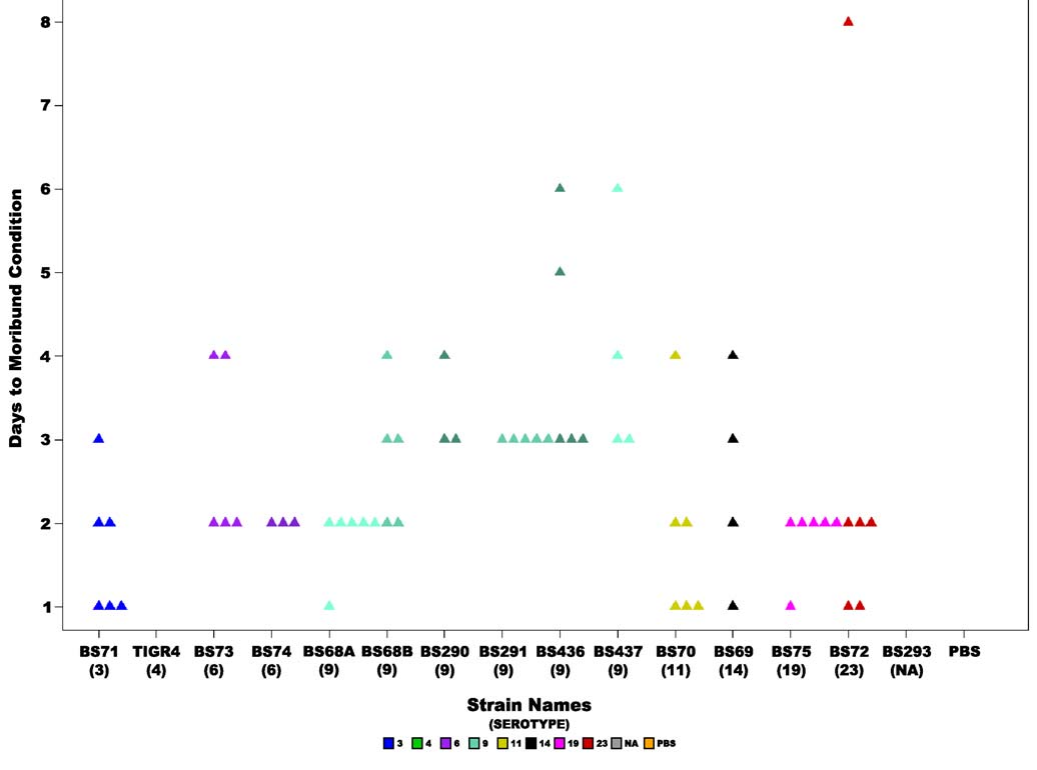
Days to moribund condition. Scatter plot illustrating the timing to the development of a moribund condition for each of the fourteen pneumococcal strains in the chinchilla model of otitis media. No moribundity was observed in the cohorts inoculated with TIGR4(4) or the unencapsulated strain BS293. Each triangle represents a single animal. Colors represent different serotypes, and shades of the same color different strains of the same serotype or in the case of BS68(9) two cohorts from different experiments. NA: non applicable.

Animals that were inoculated with BS71(3) and BS72(23) rapidly displayed signs of systemic moribundity, which effected our ability to gauge the development of otoscopic disease past day 2 (Fig. 4). Importantly, many strains show otoscopic changes by day 1 or 2 (Fig. 2), demonstrating that systemic moribundity by day 2 does not prohibit the development of otoscopic signs, as is most evident for strain BS68(9), which showed high otologic scores by day 2 (Fig. 3 and 4). Nonetheless, to account for the affect of the loss of animals infected with systemically virulent strains on the strain's otoscopic scores, we performed Kaplan-Meier Survival Probability Estimates. These results confirmed that days to onset of otoscopic disease were significantly different between the strains (the log-rank test for days to onset of moderate or worse otologic disease had a p-value = 6.446e^−12^, Table 2).

### Systemic Signs

**Figure 5:**
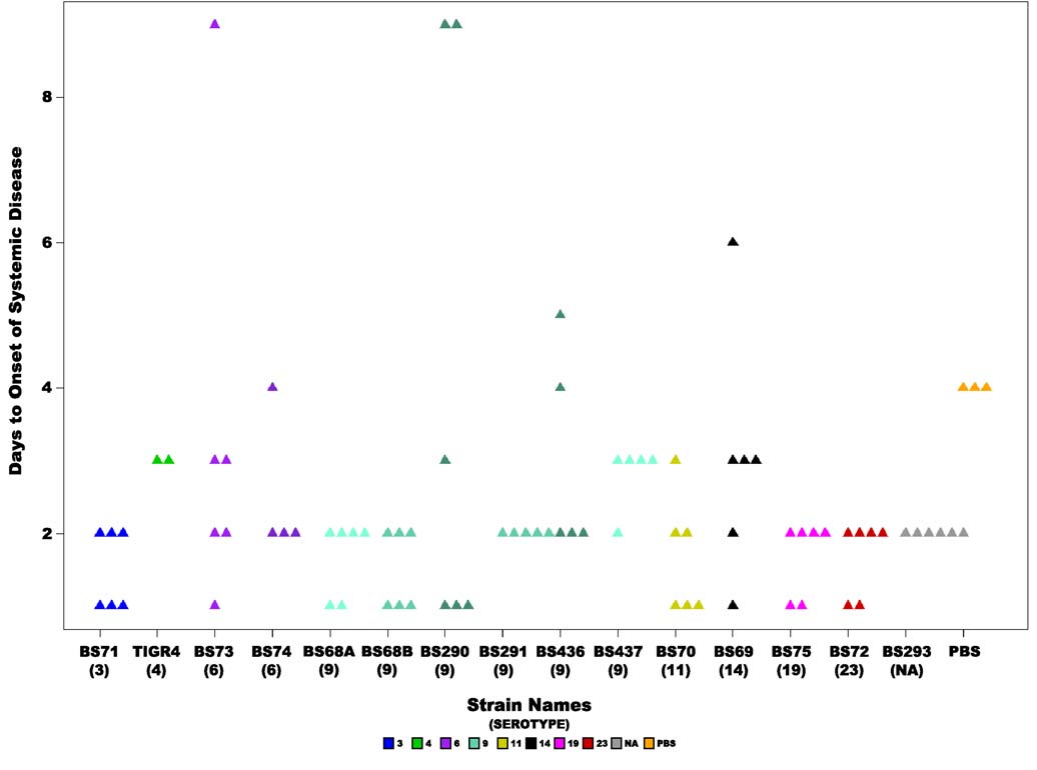
Rapidity of onset of any systemic disease signs. Scatter plot showing the number of days it took for chinchillas inoculated with each of the *S. pneumoniae* strains to develop any signs of systemic disease (score >0). Statistical analysis of all the strains did not reveal a significant difference among them. Each triangle represents a single animal. Animals are not included that did not develop disease before the end of the experiment. Colors represent different serotypes, and shades of the same color different strains of the same serotype or in the case of BS68(9) two cohorts from different experiments. NA: non applicable.

**Figure 6:**
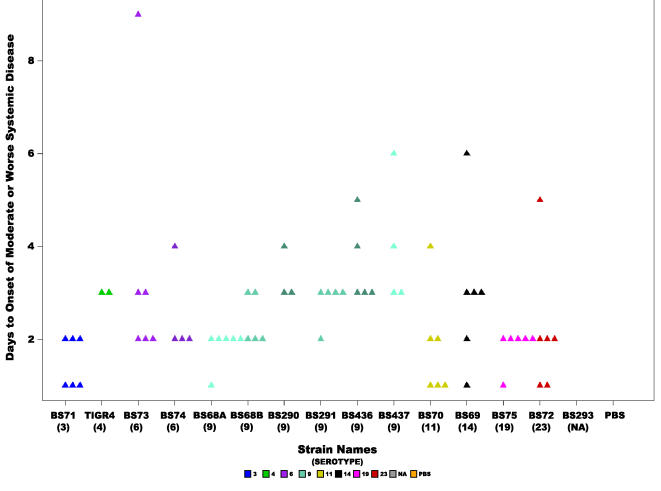
Rapidity of onset of moderate or worse systemic signs. Scatter plot showing the number of days it took for chinchillas inoculated with each of the *S. pneumoniae* strains to develop moderate or worse signs of systemic disease (score ≥2). Statistical analysis of all the strains revealed a significant difference (ANOVA p-value = 0.03 and Kruskal-Wallis p-value =  0.00118). Each triangle represents a single animal. Animals were not included that did not develop systemic disease before the end of the experiment. Colors represent different serotypes, and shades of the same color different strains of the same serotype or in the case of BS68(9) two cohorts from different experiments. NA: non applicable.

The rapidity of the development of systemic signs across all fourteen strains produced a mean of 2.3 days±1.6 (Fig. 5). Table 1 describes the signs of systemic disease for which the animals were monitored, scores of 0.5 and above were considered, and animals that never developed systemic signs were excluded from the analysis. The means ranged from 1.5 days for strain BS71(3) and 1.58 days for BS68(9) to 4 days for BS290(9) (Table 3). Analysis of this data using ANOVA does not show any statistical significance amongst the strains, however, repetition of the analysis by the non-parametric Kruskal-Wallis test yielded a statistically significant p-value of 0.016 (Table 2). Since even healthy animals showed slightly lethargic behavior (score 0.5), as evident by the PBS control, we also analyzed the days to onset of moderate or worse systemic disease (only scores of 2 or above; thus all animals that did not develop at least moderate symptoms were excluded from this analysis) (Fig. 6). These values range from 1.5 days for BS71(3) to 4 days for BS437(9) (Table 3). The results showed a significant difference amongst all strains (ANOVA p-value = 0.03, Kruskal-Wallis test p-value =  0.001) (Table 2).

**Figure 7:**
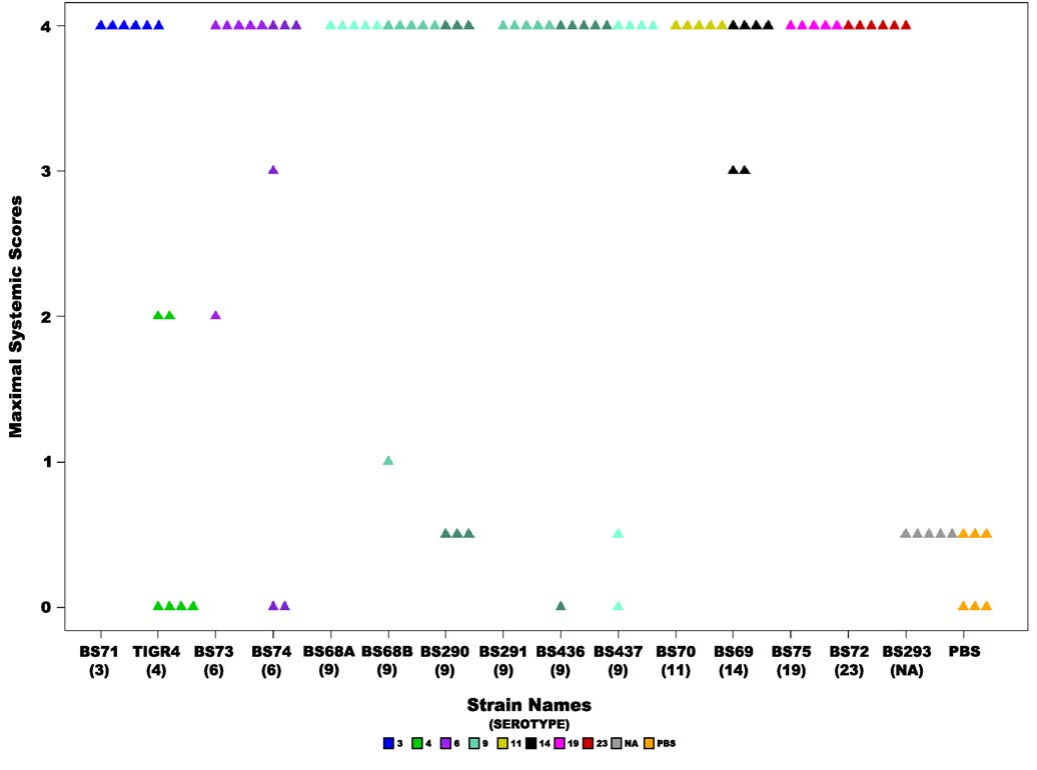
Severity of systemic signs. Scatter plot illustrating the maximal systemic score for each animal inoculated with each of the *S. pneumoniae* strains, where each triangle represents a single animal. Statistical analysis of all the strains revealed a significant difference (ANOVA p-value = 2.69e^−9^ and Kruskal-Wallis p-value = 4.2^−6^). Colors represent different serotypes, and shades of the same color different strains of the same serotype or in the case of BS68(9) two cohorts from different experiments. NA: non applicable.

The maximum systemic severity score was highly significantly different amongst strains (ANOVA p-value =  2.69e^−9^ and Kruskal-Wallis p-value = 4.22e^−6^)(Table 2, Fig. 7). The mean score for all fourteen strains was 3.11±1.52. It ranged from 0.5 for the unencapsulated strain BS293 (as well as the PBS control) to 4 for strains BS291(9), BS75(19), BS71(3), BS72(23), and BS70(11), where all animals demonstrated moribundity (Table 3). Tukey-HSD analysis of all the strain pairs showed that the unencapsulated strain BS293 and TIGR4(4) differed significantly from BS291(9), BS436(9), BS68(9), BS69(14), BS70(11), BS71(3), BS72(23), BS73(6), BS75(19), in addition BS293 also differed from BS437(9).

There was a significant correlation between severity of otoscopic disease and systemic rapidity (Spearman's rank correlation rho = 0.42 with p = 1.9e^−4^), and between severity and rapidity of systemic disease (Spearman's rank correlation rho = −0.30 with p = 5.95e^−3^); while there was no significant correlation between otoscopic and systemic severity.

Unencapsulated strains are not thought to cause systemic disease, thus we repeated the systemic score statistical analyses using only the capsulated strains (Table 2). Systemic severity scores were also highly significantly different amongst the encapsulated strains (ANOVA p-value  = 1.22e^−5,^ and Kruskal-Wallis p-value = 0.000171). Note that the onset of moderate or worse systemic disease has the same p-value as amongst all strains, since the unencapsulated strain never reaches moderate or worse disease and thus was already excluded from this analysis.

#### Strain Specific Moribundity

The moribundity of the chinchillas differed significantly amongst the strains, as determined by Fisher's Exact test (p-value = 3.9e^−7^) (Table 2). Strains BS291(9), BS70(11), BS71(3), BS72(23), and BS75(19) were the most lethal, producing 100 % moribundity within the cohorts; in contrast, no animals inoculated with TIGR4(4) and BS293-nonencapsulated reached a state of moribundity (Table 4). Again, since unencapsulated strains are not thought to cause systemic disease the Fisher's Exact test was repeated amongst just the capsulated strains, confirming that there are major differences in moribundity among the encapsulated strains (p-value = 0.000117).

**Table 4:** Strain Specific Moribundity.

Strain	BS290	BS291	BS293	BS436	BS437	BS68	BS69	BS70	BS71	BS72	BS73	BS74	BS75	TIGR4
Serotype	9	9	NA	9	9	9	14	11	3	23	6	6	19	4
**Moribundity**														
FALSE	3	0	6	1	2	1	2	0	0	0	1	3	0	6
TRUE	3	5	0	5	4	11	4	6	6	6	5	3	6	0

Fisher-Exact Test comparing all strains: p-value = 3.9e^−7^ and comparing capsulated strains: P-value = 0.000117.

NA: Not Applicable

#### Intra-serotype Specific Statistical Tests

We evaluated five independent serotype 9 strains (BS68, BS290, BS291, BS436, and BS437) to determine if the entire range of clinical phenotypes observed among the 9 strains, each with a unique serotype, would be present within a single serotype as a test to determine if total genotype (as opposed to serotype alone) played an important role in disease phenotype.

Analysis of the rapidity of otoscopic changes showed a significant difference among the five serotype 9 strains (ANOVA p-value =  0.008, Kruskal-Wallis p-value = 0.01) (Table 2). The mean was 1.6 days±0.6, and the individual strain means ranged from 1 for BS291 to 2.16 for BS436 (Fig. 2, Table 3). No statistical differences were observed in otoscopic severity among the five serotype 9 strains (Table 2), which had a mean score of 2.45±0.65.

There were no significant differences amongst the serotype 9 group for rapidity of onset of systemic disease, the mean was 2.48 days±1.9 (this is not surprising since this score was also not significant amongst all strains) (Fig. 5). In contrast, the onset of moderate or worse systemic disease is significantly different amongst the serotype 9 group (ANOVA p-value = 0.001 and Kruskal-Wallis p-value = 0.0017) (Table 2, Fig. 6). Finally, the difference for systemic severity scores amongst serotype 9 strains was not significant (ANOVA p-value = 0.17 and Kruskal-Wallis p-value = 0.18). The mean score was 3.28±1.4, which ranges from 2.25 for BS290 to 4 for BS291 (Table 3 and Fig. 7).

## Discussion

The chinchilla OM model provides a useful and facile system to study the spectrum of disease induced by multiple clinical *Sp* strains of unique genotype. In the current study we used an objective clinical scoring system [38] to investigate the differences in virulence induced by a collection of *Sp* strains that included 8 of the serotypes most frequently associated with OM, including multiple isolates of serotype 9. These data established that individual strains, even within a single serotype, have a wide variation with regard to their ability to induce both local and systemic disease, and that there is little if any linkage between a strain's propensity to induce local and systemic signs, most evident by TIGR4(4) that induced strong local disease but very mild systemic changes. The variation amongst animals within cohorts was lower than the variation amongst all cohorts suggesting that the bacterial strain type plays a major factor in pathogenesis, however, the genetic background of the host is also clearly important as there is variability of disease within a cohort of animals infected with a single isolate. It is important to remember that the chinchillas used in these studies are of outbreed stock and are therefore genetically heterogeneous. Thus, they would not necessarily be expected to have a stereotypic host response.

An example of the *Sp* strain-specific effects can be seen by the large difference in local effects induced by BS71(3) and BS72(23) relative to BS75(19), BS437(9), BS73(6), BS69(14) and TIGR4(4). Similarly, the strain-specific systemic effects were highly significant when comparing strains BS70(11), BS71(3), BS72(23), BS75(19), and BS291(9) with TIGR4(4) and unencapsulated BS293. Strains BS437(9) and TIGR4(4), which caused significant local disease, were only average or mild in their systemic virulence, respectively. Similarly, some strains that produced rapid, fulminant systemic signs (strains BS70(11), BS71(3) and BS72(23)) did not cause clinically important local disease. This dichotomy may be due to one or more of several factors. However, it is likely that a major contributor to the observed differences in disease results from the unique combination of virulence genes that each strain possesses [29], [31]. The clinically different disease states presented by several strains, with the same serotype (9), strongly suggest that configuration of the capsular polysaccharides that confer serotype may not always play a major role in the disease process. Interestingly, the unencapsulated strain BS293, which causes significant localized disease, was obtained from the same patient at two separate clinic visits 23 days apart suggesting that this is a persistent unecapsulated strain (manuscript in preparation, Hiller, Hu, and Ehrlich).

We have recently completed whole genome sequencing on 11 of the 13 clinical strains evaluated in this study, which revealed very extensive genomic plasticity among them [31] and unpublished data. After annotating each genome, a conservative gene clustering algorithm was used [39] to bin together orthologous genes from the different strains to ensure that simple allelic difference did not result in our calling orthologous loci as unique. The gene clustering results were then used for the performance of global (all strains together) genome-wide comparisons to determine the number of core genes, those present in all strains, as well as the number of distributed and unique genes present in each strain and collectively within a 24 strain supragenome. These analyses were followed by the performance of an exhaustive set of pair-wise genic comparisons across all strains, which showed that on average each strain varied from every other strain by the gain or loss of nearly 400 gene clusters. Since on average each strain pair only contains 1795 gene clusters in common this means that ∼18% of each genome is unique with respect to every other genome on average. Included among the distributed and unique gene sets for each of these strains were a large number of candidate virulence genes, many of which have not been previously identified among the pneumococci [29]. One such example includes a cluster of 10 adjacent genes that are always found together, such that if a strain possesses one of them it possesses all of them suggesting that these genes may represent a previously unrecognized mobile genetic element particularly as one of the genes annotates as an integrase. This gene cluster is possessed by all of the highly virulent strains including BS70(11), BS75(19), BS72(23), BS71(3), BS291(9), and is also found in one of the intermediately virulent strains, BS68(9), as well as one of the less virulent strains BS290(9), but is absent among the remainder of the avirulent and intermediately virulent strains including TIGR4(4), BS74(18), BS69(14), and BS73(6). These genes are also present in the systemically avirulent unencapsulated strain BS293, but this strain's lack of systemic virulence is attributable to its lack of capsule. Five of these ten genes are organized into one operon and have been annotated as being involved in cellobiose PTS transport and metabolism. This is interesting because McKessar and colleagues (2007) previously showed that disruption of one of the virulence-related two-component systems in Sp, TCS08, resulted in suppression of a putative cellobiose transport system (PTS)[40]. The PTS system we have identified is only distantly related to the one described by McKessar et al. with some of the components having very limited identity (30–40%) and others which show no measurable identity whatsoever. Nonetheless, their results suggest that PTS cellobiose systems may play a role in virulence; making the PTS system described here an interesting candidate for contributing to Sp systemic virulence based upon its segregation largely within the more highly systemically virulent strains. Together these genomic and phenotypic studies suggest that the virulence factors for local and systemic disease may be unique and that some strains have accumulated more of one class than another, whereas some strains have accumulated genes from both classes. Moreover, it is clear that serotype replacement occurs amongst Sp strains, suggesting that strains that are very genetically diverse may share the same serotype, likewise strains that are highly related may have different serotypes. We have observed strain evolution in vivo that has resulted in as much as 20% genome replacement by a donor strain (Hiller et al. unpublished observations). Genome sequencing of multiple Sp strains confirms this, since strains of the same serotype are often spread apart in a phylogenic comparison based on genic differences [31]. This enormous plasticity of Sp strains makes it difficult to predict across any length of time or within different geographic regions what the pathogenicity of any specific strain or serotype will be.

The observation that there is extensive genomic plasticity among *Sp* strains does not preclude the contribution of unique host factors to clinical phenotype and outcome. Such host factors could include differing expression levels of certain immune system components that may affect susceptibility or resistance to certain pathogens [10], [41], [42].

Induction of OM in the chinchilla results in a reproducible model of local infection that reliably mimics the clinical disease. The dichotomy between a strain's proclivity to cause local and systemic disease is entirely consistent with the spectrum of human disease caused by *Sp*. The clinical challenge remains to differentiate those patient-strain combinations that will result in infectious spread by invasion or dispersion, from those that will have self-limited disease. This model provides the unique opportunity to separate pathogen weaponry from host response.

## Materials and Methods

### Bacterial strains and culture

Thirteen pneumococcal strains (all ampicillin sensitive) were obtained as nasal washes from symptomatic pediatric participants at Children's Hospital of Pittsburgh who were enrolled in a Fluzone vaccine trial. All clinical *S. pneumoniae* strains were isolated and restreaked for single colonies on Trypticase™ Soy Agar (TSA II) supplemented with 5% sheep's blood (Becton Dickinson, Sparks, MD), and then cultured in Todd Hewitt broth (Sigma, St. Louis, MO) at 37°C in a humidified 5% CO_2_ atmosphere for one passage followed by aliquotting and cryopreservation in 22% glycerol at –80°C. Pneumococcal isolates were typed by the Quellung reaction using polyvalent serogroup and serotype-specific antisera (Statens Seruminstitut, Copenhagen, Denmark). The eleven strains, designated as BS68-75, BS290, BS291, BS436, BS437, BS293, were serotyped as types 9V, 14, 11, 3, 23F, 6A, 6A, 19F, 9V, 9V, 9V, 9V and nontypeable, respectively. The TIGR4 (BS90, serotype 4) strain was also obtained and plated. Eleven of the thirteen (excluding BS436 and BS437) clinical strains have been completely sequenced to an average depth of coverage of >20-fold and have been demonstrated to be unique with respect to their genic content [29], [31]. Middle-ear inocula for each strain were prepared by plating one of the first passage aliquots on blood agar for overnight incubation. Chinchillas were inoculated bilaterally with ∼100 CFU/ear based on cultures with an OD_A600_ of 1 of having 5×10^8^ CFU/mL. The actual inoculating titers were determined by standard dilutional plate counts.

### Animal experiments and monitoring

All experiments were conducted with the approval of the local Institutional Animal Care and Use Committee (IACUC). Research grade young adult chinchillas (*C. laniger*, 400–600 gm; McClenahan Chinchilla Ranch, New Wilmington, PA) were obtained free of middle-ear disease. After a protocol-directed period of environmental acclimation, induction of anesthesia was attained on day 0 by intramuscular injection of 0.1 mL of a solution of ketamine hydrochloride 100 mg/mL, xylazine hydrochloride 30 mg/mL and acepromazine 5 mg/mL. After anesthesia was confirmed (abolishment of the eye-blink reflex), 0.1 mL of a 1000 CFU/mL *Sp* culture was injected bilaterally into the tympanic bullae using a 0.5 in, 27-gauge needle attached to a 1 ml syringe (Fig. 1). Each of the fourteen pneumococcal strains was used to infect a cohort of 6 chinchillas. BS68 was inoculated into two cohorts of 6 chinchillas to ensure experimental consistency across temporally distinct inoculation groups; the data from the two BS68 groups were combined for statistical analysis. Importantly, there was no statistically significant difference between the two BS68-inoculated sets using both parametric and non-parametric tests. The BS291 cohort was reduced to 5 due to a death unrelated to the experiment.

A multiparameter scoring system based on signs of local and systemic disease (Table 1) was applied to each chinchilla on a daily basis for ten days to assess the degree of disease induced by the individual pneumococcal strains. All clinical evaluations were performed by an otoscopist who was blinded with respect to the strains that had been used to inoculate each animal. This scale has demonstrated utility in clinical scoring of the chinchilla's response to direct bacterial inoculation of the bullae [38]. A control cohort of 6 animals was inoculated with sterile phosphate buffered saline. Moribund animals (having a systemic severity score of 4) were either treated with 150-mg/kg ampicillin daily (all *Sp* strains were ampicillin sensitive) or sacrificed in accordance with the IACUC protocol. Once an animal reached this state evaluation ceased, as moribundity was considered an end point.

### Statistical Analysis

Raw scores for local and systemic disease ranged from 0 (clinically normal) to 4 (extreme severity) (Table 1) and moribundity was recorded (Table 4). Results are reported as mean±standard deviation (SD). One-way ANOVA and Kruskall-Wallis tests were utilized in comparisons across cohorts to determine if the differences in local and systemic diseases were statistically significant among strains. Tukey's HSD test was utilized for pairwise comparisons between strains. The Fisher Exact test was utilized to examine the statistical significance of moribundity data across all cohorts (Table 4). Kaplan Meier estimates were utilized to perform log-rank tests that measure for strain differences in local disease onset. Significance was defined as *p-value* ≤0.05. The open source software package R was utilized for all statistical analysis (http://www.r-project.org/).
